# Book Reviews

**Published:** 1822-05

**Authors:** 


### Monthly Medical and Scientific Bibliography.

Vexat censura . . . . . . Corvos.

### 1. GREAT BRITAIN.

**Art. I.**—*Medicamina Officinalia, seu Pharmacopœiæ Londinensis, Index Methodicus.* Cura F. A. Macann, m.d. 18mo. pp. xii. 93. Burgess and Hill, London. 1822.

We cannot convey a better idea of this useful little volume, than by quoting the author's own words respecting the necessity of it, and his explanation of its plan and construction.

"1. When officinal medicines* are considered with reference to their employment in the cure of diseases, they naturally divide themselves into two classes, one of which consists entirely of articles derived or formed from the other.

"2. To the articles thus derived or formed, the name of Preparations is usually given; while the others are, in contra-distinction, often denominated Simples.

"3. An accurate knowledge of the different relations existing between these two classes,—that is, a knowledge of all the preparations which exist of any particular simple,—is a matter of much importance to the practitioner.

"4. This knowledge, however, is not to be acquired from the Pharmacopœia, but at the expense of much time and labour; for in that work, generally speaking, the articles are arranged as if they were entirely unconnected of each other.

"5. This is a defect inseparable, perhaps, from the plan upon which the Pharmacopœia is constructed: it is a defect, however, for which a remedy may be found in a 'Digested Index;'—such an Index I have attempted, and now submit to the public.

"6. I shall not here enter into any discussion relative to the principles upon which this Index is founded, for such discussion would, in my opinion, be misplaced: avoiding, therefore, all unnecessary detail, I proceed at once to explain the plan and construction of the work.

"7. I have stated (1, 2,) that officinal medicines are divisible into two classes, one denominated Simples, the other Preparations; that is, preparations of those simples.

"8. Of some simples many preparations exist, of others none at all, but no preparation can exist without an antecedent simple: in arranging the simples, therefore, we arrange the whole; for every preparation will naturally find a place under that simple from which it is derived.

"9. The plan, then, of this Index is—

* "By 'officinal medicines' I mean those contained in the London Pharmacopœia, to which work, on the present occasion, I strictly confine myself: the edition always referred to is that of 1817, in 18mo."

"*a.* To exhibit all the officinal simples in one continued alphabetic series;

"*b.* To enumerate, under each simple, all those articles which are in any manner derived or formed from it."

"11. Thus arranged, the simples may, with strict propriety, be denominated *leading articles*, while the others may, with equal propriety, be denominated *subordinate articles*; and these terms, simply expressive of a fact, we shall in future employ, in preference to the others which involve matter of opinion."

The class of *leading articles* contains—

*a.* The officinal plants and animals, and animal and vegetable substances.

*b.* The acids, alkalies, earths, and metals, to which the author adds *sulphur*.

*c.* And also alcohol, aqua distillata, carbo ligni, fermentum, petroleum, sapo durus, sapo mollis, succinum, vinum.

To each leading article, alphabetically arranged according to their officinal or systematic name, there is subjoined its English name.

The class of *subordinate* articles embraces every article not included in the preceding class.

Opposite to each article there is a marginal number, referring to the pages of the 18mo. edition of 1817 of the London Pharmacopœia.

There is also an Appendix, containing notes of the author on some of the articles, exhibiting considerable judgment, and a tolerable share of erudition.

Bound up with the Pharmacopœia, to which the present volume is literally *une table des matières raisonées*, Dr. Macann's humble performance will be found of the greatest utility.

It would be well for the future compilers of our national Pharmacopœia, if they were to attend to several very sensible suggestions and hints to be found in Dr. Macann's notes.

---

Art. II.—*A Pharmaceutical Guide: in two Parts.—Part I. A Latin Grammar, in which all the Rules are illustrated by Examples selected from the London Pharmacopœia.—Part II. An Interlineary Translation of such Formulæ in the London Pharmacopœia as have been found difficult to be comprehended by some young Medical Students. To which is prefixed, a Vocabulary of Words most frequently employed in Prescriptions; with Examples of their Use.* By the Author of the Student's Manual, an Etymological and Explanatory Vocabulary of English Words derived from the Greek. 12mo. pp. 92. Longman and Co. London. 1822.

There have always been, and are even at this moment, persons who affect to despise the Latin tongue,—who consider it useless in medicine,—and who could wish it were altogether expunged from every system of education. The members of the Constitutional Assembly, in the early part of the French revolution, decreed the total extinction of the Roman idiom, as one which was more adapted for aristocratic than republican intercourse; and, also, because the flourishing days of the Latin tongue had been those of incipient despotism. From that time the classical language of Latium has formed but a slender part of the education of children in France: few of those who apply themselves to medicine have studied Latin, or have any idea of grammar. Much the same observation applies to a large proportion of those who are brought up to physic in this country. Until very lately, when a provision was enacted by the Apothecaries' Company to render some knowledge of Latin necessary, scarcely one general practitioner out of fifty understood that idiom. As for the surgeons, no collegiate body has ever troubled itself about its members being, or not, acquainted with Latin: nor can it be said, that the higher branches of the profession have taken great pains in making themselves masters of a language in which the most eminent physicians of all ages have composed their valuable works, or delivered their instructive *prolusiones*.

It is no rare occurrence to hear of some medical man, of high pretensions and some claims to public consideration, being rejected, now and anon, by the president and censors of the Royal College of Physicians, because unable to comprehend or to speak the Latin tongue. Nor is it unusual to meet with practitioners, whose illegible and ill-abbreviated prescriptions betray the grossest ignorance of the language in which they affect to write.

If this be a correct view of the state of classical education among the different branches of our profession, (and we are sure that many will assent to our proposition,) there can be no doubt but that the work before us must be calculated to do much good, if properly consulted by those individuals for whom it is purposely written.

The work embraces the rudiments of a medical Latin grammar, and an interlineary translation of such formulæ in the London Pharmacopœia as have been found difficult to be comprehended by some young medical students. The author has, moreover, added a vocabulary of words most frequently employed in prescriptions, with examples of their use.

Of the first part, relative to the medical Latin grammar, we do not think it necessary to say any thing, except that it is correctly, and, we may add, ingeniously displayed: and as, without some knowledge of grammar, no translation can enable a person wholly ignorant of Latin to understand thoroughly what is written in that language, it becomes an object of great convenience to have the elements of such a knowledge by the side of the very difficulties which those elements are calculated to explain.

With regard to the second part, containing the translation of the London Pharmacopœia, we wish to speak more distinctly in its favour. To give the meaning of each word as nearly as possible, was the principal object aimed at by the author: elegance of expression was not sought for; and, when collated with two other translations of the Pharmacopœia which have been published, the one now brought forward will claim a priority of commendation from the student.

The following is an example of the interlineary and *verbatim* translation of the directions given by the College of Physicians for preparing the carbonate of magnesia; and may be taken as a fair specimen of the manner in which the translation is executed.

"MAGNESIÆ CARBONAS.

"Potassæ subcarbonatem in aquæ octariis tribus, magnesiæ
Of potash the subcarbonate in of water pints three, of magnesia
sulphatem in aquæ octariis quinque, separatim liqua, et cola; dein
the sulphate in of water pints five, separately dissolve, and strain; then
liquori magnesiæ sulphatis reliquam aquam adjice, et
to the solution of magnesia of the sulphate the remaining water add, and
coque, ei-que, dum ebullit, liquorem priorem admisce, spathâ
boil, it-and, whilst it boils, the liquor former mix, with a rod
assiduè movens; tum per linteum cola; denique pulverem, affusâ
constantly stirring; then through linen strain; lastly, the powder, being poured on
sæpius aquâ fervente, ablue, et calore gradûs ducentesimi
frequently by water hot, wash, and with a heat of the degree two-hundredth
super chartam bibulam exsicca."
upon paper blotting dry."

The vocabulary of words most frequently employed in prescriptions, is subdivided into what relates to the nouns and verbs, and to the use of both these in phrases. We shall conclude this article with a short extract from each of those subdivisions of the volume.

"NOUNS.—Alvi, *gen. of* alvus, *masc. and fem.*—alvo adstricto—'the bowel being confined:' here 'alvus' is masculine, but in the following phrases it is feminine,—donec alvus soluta fuerit—ad alvum officii immemorem excitandam—post alvum exoneratam. We find also —alvo astrictâ—('adstringo' is used by some writers for 'astringo.')

"Haustûs, *gen. of* haustus, *mas.*—haustus sumendus, *or* dandus, *or* deglutendus, *or* hauriendus, *or* capiendus—pro haustu salino—haustus diaphoreticus—pro haustu aperiente—haustus opiatus—superbibendo haustu sequente—pro haustu emetico—deinde capiat æger haustum aliquem purgantem."

"VERBS.—Illineo—illinatur embrocatio—cujus illinatur in partem affectam.

"Impono — imponatur emplastrum lyttæ — cataplasmata pedibus imponenda.

"Ineo— ineunte paroxysmo.

"Infesto—acido infestante.

"Infrico—embrocatio ventriculi regionem infricanda.

"Injicio—injiciatur hora somni—sæpissimè injiciatur paululum inter oculum et palpebras.

"Inspergo—cujus inspergatur pauxillum super mamillas.

"Invadeo—invadente paroxysmo."

"Phrases.—*Syrupi quantum sufficit ad acorem compescendum, et gustum conciliandum; sumat quotidie instar potus, et bibat quantum sitis exigat*—As much syrup as suffices to diminish the tartness and to gratify the taste, (or, please the palate;) let him take (it) daily like a common drink, and let him drink the quantity thirst may require.

"*Simul benè contritis, sit emplastrum scuto pectoris*—Being rubbed well together, that there may be a plaster for the defence of the breast, (that is, the chest.)

"*Fiat linctus, de quo sumatur cochleare parvum nocte maneque, cum haustu cujusvis potus tenuioris tepefacti*—Let a linctus be made, of which let there be taken a small spoonful night and morning, with a draught of any thin drink made hot.

"*Initio sumat æger pilulam unam pro dosi mane ac nocte, postea sumat binas, dein tres, et denique augeatur dosis quantum fieri potest*—Let the patient take, in the beginning, one pill for a dose, morning and night; afterwards let him take two, then three; and, lastly, let the dose be increased as much as can be done."

At the same time, we admit, that the selections of phrases of our author might have been made from among those of more elegant Latin writers, of which, an abundant opportunity was afforded to him by the continental authors: and we are free to confess, that there are some few errors, which it would be well to correct.

---

Art. III.—*The New Medico-Chirurgical Pharmacopœia; being a Selection of Modern Formulæ, from the Private and Hospital Practice of the most eminent Members of the Profession, in Europe and America: for the use of Surgeons and Surgeon-Apothecaries.* By a Member of the Colleges of Surgeons of London and Edinburgh. 12mo. pp. 148. Simpkin and Marshall, London. 1822.

It bespeaks a lamentable degree of prevailing superficiality in the medical education of the present day, and a deplorable deficiency in the rising generation of "healers and doctors," to see the vast number of cheap and popular trifles which daily issue from the press, for the purpose of facilitating, not the study, but the "humbug" of practice. We have, within the space of the last four pages, examined no fewer than three works, commendable in themselves, from the manner in which they are executed, and for the honest intention of their authors; which, however, are, solely, directed to make a doctor of threads and patches, and gloss over the half-finished picture so as to impose, satisfactorily, on the public. One of the works in question is to teach these gentlemen how to understand the Pharmacopœia of London ; another is to instruct them how to avoid blunders in writing their prescriptions; and a third is intended to supply them with ready-made prescriptions, to save them the trouble of thinking for themselves, (much to the benefit of their patients.)

Of the latter description is the volume before us, which, we are told, has been compiled for the exclusive use of surgeons; and the design of which is to supply the chirurgical practitioner with a complete collection of such formulæ as, in the course of his professional engagements, he may stand in need of.

To the formulæ strictly applicable to surgery, the author has found it necessary to add such remedies of the physician, as the customs of medical surgery may be supposed to warrant. Those formulæ which are directly taken from the College of Physicians, have a distinguishing mark affixed to them. The more operose of these, as the chemical preparations, are merely named, and their use, in a concise way, pointed out. The mere pharmaceutical compositions, as the collyria, liniments, unguents, &c. and their mode of application, are given somewhat in detail.

It can scarcely be expected that we should enter into an analysis of a work which admits of none. As specimens of the manner in which this compilation is drawn up, of its style, and utility, we extract, almost at random, the three following prescriptions, with the *addenda* of the author.

"ELECTUAR. PIPERIS COMPOSITA (ITUM).

"℞. *Piperis nigri, Radicis enulæ campanæ sing. lib.* j. *Seminum fœniculi dulcis lib.* iij. *Mellis despumati, Sacchari purificati sing. lib.* ij.—The three first ingredients are to be finely powdered, and well mixed; after which, the honey and sugar, melted together over the fire and formed into a clear syrup, are to be added, and the whole beaten together into a mass.

"The bulk of a nutmeg of this is ordered to be taken morning, noon, and night; drinking a glass of water or white wine after it.

"This is the celebrated paste of Dr. Ward, which, though a singular kind of composition, has so long stood the test of experiment, as certainly to deserve a place amongst our chirurgical formulæ.

"There can be no doubt of its usefulness in some diseases of the rectum; those especially which happen in debilitated habits, and which have been of long duration. In the piles, and in some cases of fistula, there are numerous proofs of its efficacy.

"When piles are attended with erysipelatous inflammation, which is a common occurrence, or are disposed to inflame, or are attended with a considerable degree of irritation, this composition generally proves very hurtful. In leucophlegmatic habits, and when piles arise from, or are accompanied with, deficiency of secretion from the internal surface of the rectum and colon, a stimulating composition generally proves beneficial.

"LOTIO ACIDI PRUSSICI.

℞. *Acid. prussic. drach.* j. *Aq. flor. aurant unc.* vj. *Misce.*—This lotion has lately been much recommended for herpetic and other inflammatory affections of the skin, and for ring-worm. It is a potent poison, and, being sold of different degrees of concentration, it should be used with great caution. It is, unquestionably, the most powerful sedative that has been discovered.

"LOTIO ACIDI PYROLIGNICI.

℞. *Acid. pyrolignic. purif. fort. unc.* j. *Alcohol unc.* iv. *Aquæ puræ unc.* iij. *Misce.*—This lotion, after abstraction of blood by leeches, has been found very beneficial in dispersing inflammation of the mammary glands during suckling. The breast should be covered with folds of soft linen, moistened with it, and re-wetted with it when it becomes as warm as the body."

In order to render his work still more useful, the author has arranged the different remedies mentioned in the volume, in a table, under the head of each of the diseases for which those remedies have been, usually, recommended. This is to make physic cheaper than ever; and we must protest against all attempts to teach one of the most noble, as well as the most difficult, of the learned professions, by means of "Pinnock's Catechisms" and twopenny playthings.

---

### 2. FRANCE.

Art. IV.—*Administration des Hopitaux, Hospices civils, Secours à Domicile, Direction des Nourrices de la Ville de Paris, et Enfans-Trouvés du Department de la Seine. Compte Sommaire de l'Exercice* 1820, *au* 31 *Mars* 1821. 4to.

It is perfectly true, that more good is done, with less ostentation, in this than in any other country; but it is likewise true, that much more good might be effected were we a little more ostentatious. Why, for instance, are not the results of hospital practice, whether in a medical or an economical point of view, made public from time to time? If there be nothing in those results to be ashamed of, they ought, unquestionably, to be published; and still more ought they to be published, if they happen to contain any thing that shuns day-light. In the former case, we should gain, by circulation, the excitement of the best feelings of philanthropy, and the approbation of the good. In the second case, we should gain by the opportunity afforded of correcting abuses, checking roguery, exposing ignorance, and reforming errors.

It is thus that the French government, who pay for the support of most of their metropolitan hospitals, and who, consequently, have a great stake in these matters, think respecting the publication of reports like the one now lying before us: nor can it be considered impertinent in us to desire that so excellent an example may be followed by those whose purses are ever open for the relief of the infirm poor in this country. They have a right to call on the civil and medical officers of the charitable establishments they contribute to support with their money, to lay open the machinery of their practice, and to acquaint the public at large with the result of their exertions.

The present French hospital-report embraces a period of fifteen months, and extends to the consideration of the revenue, the expence, the population of, and the comparative results in, the different hospitals in Paris. The report is published by the council of general administration of the hospitals, composed of several illustrious individuals, mostly peers of France, who act gratuitously.

There is, moreover, a directing committee, the members of which are paid, and who have the immediate superintendence of the hospitals.

The different establishments considered in the present report are hospitals, *hospices*, or *asylums*, and their dependencies, *bureaux de charité*, and the establishment for foundlings. We can only give a rapid view of the

GENERAL RESULTS.

| | Francs. | Cents. |
|---|---|---|
| 1. REVENUE.—*a.* Fixed and variable income of each establishment under the immediate direction of the council | 3,903,457 | 09 |
| *b.* Sums granted by government on different funds | 5,764,000 | 0 |
| *c.* Casual receipts | 126,012 | 0 |
| | 9,793,469 | 09 |
| 2. EXPENDITURE.—*a.* Hospitals, hospices, and their dependencies | 6,278,640 | 71 |
| *b.* Bureaux de charité | 1,770,604 | 93 |
| *c.* Foundlings | 1,282,806 | 98 |
| *d.* Contingencies | 368,229 | 93 |
| Total Expenditure | 9,700,282 | 55 |
| Balance in favour of the revenue | 93,186 | 75 |

| | | |
|---|---|---|
| 3. Population.—*a.* | Hospitals, hospices, and their dependencies | 62,682 |
| *b.* | Foundlings | 17,349 |
| *c.* | Clinical establishment | 913 |
| *d.* | Other establishments | 2,822 |
| *e.* | Out-door patients | 82,643 |
| | Total relieved or admitted | 166,409 |

4. Comparative Expenditure.—The amount of expenditure during the period comprised in the present report, and for each person relieved or admitted, is 1 franc, 16 centimes, and 29 deniers.

There is matter in this report for a very long and instructive article; but the limits of our publication oblige us to be satisfied with giving only the above very superficial outline.

---

3. AUSTRIA.

Art. V.—*Physiologie oder Lehre von der Natur des Menschen, &c.;* —*or, Physiology of Human Nature.* By Professor G. Prochaska. 8vo. pp. xiv. 608. Vienna.

In the introduction to this work, the author has taken into consideration those forces by which every animal phenomenon seems to be produced; and he is inclined to think that, although some of the physiological functions seem influenced by physical, mechanical, hydraulic, and chemical laws, animal phenomena cannot, nevertheless, be explained without some reference to electricity.

The work is divided into sections.

The first section is on the external condition of man. His origin, prerogatives, physical system, and general functions, are severally considered, as well as the variety of human races.

In the second section, we find a description of the principal parts of the human body, with some reflexions on their reciprocal connexions. Man, according to our author, consists of a spiritual and a corporeal part. The formation of the cellular, muscular, and nervous system, is fully entered into, and developed. The humours, too, of the body are considered.

The following curious proposition is discussed in the third section:—"Life is especially derived from electrical action." The electrical phenomena are described by the professor, at large, and an application made of them to the composition and decomposition of the body. The physiology of secretion is explained, on the same principle on which philosophers have endeavoured to account for the phenomena of light, heat, magnetism, &c.

Section 4th is on the nerves and their functions. Every sensation is the effect of life and stimulus. In organic life, every sensation is regulated by electric polarity. The laws which influence our sensations are those of the galvanic circle.

*a.* On the sense of *touch* he has advanced nothing new.

*b.* On *taste.* The gustative bodies are considered, by Prof. Prochaska, as links of the galvanic chain.

*c.* On the sense of *smell* we find nothing worthy of observation.

*d.* *Hearing* is explained on mechanical principles.

*e.* *Sight* depends on a gentle motion produced by the transmission of light through the cornea and humours, and propagated, by means of the nerve, to the brain.

Professor Proschaska regards the brain as the organ of thinking, although nothing in its structure may account for this peculiarity of its functions. Electricity is again referred to with a view to explain those functions.

We do not profess to understand the author's ideas on *muscular motion.* Indeed, all that we have already stated is, to us, little short of unintelligible; but we were anxious that our readers should see in what manner an eminent anatomist lost his time, and to what length the absurdity of *tudesque* philosophy has, lately, extended. Our author is of opinion that muscular motion depends, principally, on the change of the poles of muscular electricity, when stimuli are made to act on muscles!

In section the fifth we have the author's opinions on the circulation of the blood in general. He regards the minute globules described by authors as constituent parts of the blood, to be mere optic deceptions. The progress of the blood through the capillary vessels, is explained on the principle of attraction and repulsion, produced by the contact of *acid* and *carbone,* giving rise to a common galvanic phenomenon.

Section sixth treats of respiration; and the seventh on nutrition and re-production. In this latter section, the questions of hunger and thirst are amply discussed.

The two last sections are on generation and the age of man; in which latter consideration the author has closely followed his countryman PLOUQUET.

---

### 4. PRUSSIA.

ART. VI.—*De Dysphagia Commentatio Pathologica.* Auctore GUSTAVO KUNZE, M.D. Plates. 8vo. pp. 126.

THE author divides this disease into dysphagia œsophagea and pharingea, each of which is subdivided again into a variety of species.

The first section treats of dysphagia in general. In tracing the history of this disease, the author begins from the time of HIPPOCRATES, and comes down as late as the publications of BLEULAND and MATT. VAN JEANS. He arranges dysphagia into the following species:—1. Organica. 2. A strictione. 3. Ab obstipatione. 4. A compressione. 5. Dynamica. 6. Cum adaucta sensibilitate. 7. Cum congestione. 8. Cum inflammatione. 9. Cum spasmo. 10. Cum imminuta sensibilitate. 11. Cum stagnatione. 12. Cum paralysi. 13. Gangrena. 14. Sphacelo.

The second section is full of interesting points. It treats chiefly of dysphagia œsophagea. This disease begins gradually, and depends on organic causes. At first there is only a trifling difficulty in drinking, which can easily be overlooked. The food is arrested in a particular spot of the œsophagus, until, by stronger efforts of the muscles of deglutition, or with the assistance of drinking, it is either propelled into the stomach, or rejected by vomiting, by the antiperistaltic motion of the œsophagus. The *vomitus* œsophageus, which takes place without the co-operation of the diaphragm and abdominal muscles, constitutes one of the principal pathognomonic symptoms of this disease.

The author is inclined to attribute the presumed infrequency of this disease to the inaccurate observation of medical men, who often suffer it to go unnoticed.

Dr. KUNZE enters next into the consideration of what constitutions appear to be more or less liable to the disease; and concludes, that its greatest frequency is observed after the age of forty years.

The occasional causes of the disease are divided into *organic* and *dynamic*. The former are subdivided into *innatæ* and *ascitæ*. Some cases of scirrhous indurations are related; and two illustrative plates, with graphic delineations of the malady, are given.

---

### 5. BAVARIA.

ART. VII.—*Ueber die Heilkräftc des Schwazen, &c.*—*On the Healing Power of Black Pepper in Intermittent Fevers.* By Dr. RIEDMILLER, of Nuremberg.

THE author, like Dr. L. FRANK, was led, by mere chance, to use the black pepper in intermittent fevers. His first case was that of a young man, whose fever had baffled every, even the most powerful, remedy, (such as bark, opium, &c.) for the space of fourteen weeks. Having learned, from an old woman, that she had derived the greatest benefit, in a similar case, from the use of black pepper, of which she had swallowed 332 grains, he was induced to try the drug on his patient, whom he thus succeeded in a short time to cure.

In the course of more than twenty years, the author states to have administered the black pepper to upwards of 500 patients, and to all with equal success.

He, however, cautions practitioners to be certain, ere they have recourse to this remedy, that the inflammatory and gastric symptoms, attending the disease, have wholly subsided.

The form in which he has administered the black pepper, is that of pills made up with mucilage.

---

### ACADEMICAL PROCEEDINGS,

